# Comment on “Hepatitis C and the Sex Trade”

**DOI:** 10.1155/2016/1253208

**Published:** 2016-04-28

**Authors:** M. Eugenia Socías, Kathleen Deering, Julio S. Montaner, Kate Shannon

**Affiliations:** ^1^British Columbia Centre for Excellence in HIV/AIDS, St. Paul's Hospital, 608-1081 Burrard Street, Vancouver, BC, Canada V6Z 1Y6; ^2^Department of Medicine, University of British Columbia, St. Paul's Hospital, 608-1081 Burrard Street, Vancouver, BC, Canada V6Z 1Y6; ^3^School of Population and Public Health, University of British Columbia, 5804 Fairview Avenue, Vancouver, BC, Canada V6T 1Z3

We read with interest Dr. Shafran's editorial [1] on our study published in November/December 2015 issue of the journal providing a baseline assessment of engagement in the continuum of hepatitis C (HCV) care among sex workers in Vancouver [2]. Given the limited peer reviewed evidence available in this area, we believe that our research, which uses data from a large cohort of sex workers in Vancouver, makes a strong and unique contribution to start filling these gaps. Here, we address the main issues raised by the editorial.

While we agree that globally injection drug use is the major driver of HCV transmission, other routes have also been documented. Particularly relevant to our study population is the potential for permucosal transmission of HCV in high-risk sexual practices and sharing of intranasal drug paraphernalia, especially in the context of multiple partners [3].

The editorial also appears to overlook the significant limitations of risk-based testing. Research demonstrates that certain risk exposures are frequently underreported due to stigma and fear of disclosing on the part of the clients [4] or failure of ascertainment by healthcare providers [5]. “Symptomatic testing” also has important limitations given that chronic HCV is frequently asymptomatic and that ALT levels are typically within normal range in up to 40% of people with chronic HCV [6]. Importantly, overreliance on risk-based or “symptomatic testing” strategies has resulted in a large proportion of underdiagnosis of HCV, which leads to missed opportunities for optimal treatment and prevention of HCV related morbidity, mortality, and onward transmission. Consequently, HCV testing guidelines are increasingly recommending additional population-based strategies [5].

As described in our paper, diagnosis of HCV infection was based on laboratory testing. Other data (e.g., engagement in the HCV cascade) relied on self-reported quantitative data collected through surveys conducted by experienced interviewers, as it is routinely done in epidemiological research and within public surveillance systems. In addition, although our study was not designed to evaluate barriers to HCV care, we discussed potential barriers, as well as potential strategies to overcome them and improve HCV care outcomes. These recommendations are informed by prior extensive research with sex workers and other marginalized populations in our setting that characterized gaps and successful strategies to improve healthcare access [4, 7].

The editorial also places a substantial amount of the responsibility for changes in health status to be the responsibility of individuals (“in the end, adult (sex workers) must want to have better health outcomes”) [1] and correspondingly places blame on individuals for whom health status does not improve. This ignores the well-established body of literature pointing to the limited agency of individuals from marginalized populations to enact change in the face of structural and socioeconomic inequities, regardless of their own desire to do so [4, 8]. Our research and many others globally have demonstrated over more than 10 years that failure by governments and policy-makers to address systemic barriers to care (e.g., extreme violence, police harassment) has pushed sex workers away from health access and in many cases forced sex workers to prioritize their immediate safety (e.g., protections from violence) over longer-term health risks [4].

Finally, the editorial suggests that HCV treatment should be discouraged among women involved in sex work. Although no data on HCV care among sex workers are available in the literature, multiple studies demonstrate that when appropriately supported other marginalized populations, such as people who inject drugs, achieve rates of sustained virological response comparable to those achieved in the general population [9]. Further, modeling studies suggest that expanding access to persons at increased risk for transmission has the potential to significantly reduce HCV incidence and prevalence, particularly when combined with other harm reduction strategies, and importantly that this strategy is likely to be cost-effective [9]. Withholding HCV treatment from populations with the highest burden of HCV is clearly against international HCV treatment guidelines, including those from the World Health Organization (WHO) and the American Association for the Study of Liver Disease (AASLD)/Infectious Diseases Society of America (IDSA) [9]. Shifting focus away from judgmental blanket assumptions about individuals' abilities to sustain treatment and personal health goals towards strategies that could best engage and support marginalized populations in long-term care would be a far more productive as well as ethical use of resources.

Given increasing availability of highly efficacious, simpler, and better-tolerated HCV drugs there is now a real opportunity to control the HCV epidemic in Canada and abroad. Equitable access to early HCV treatment and care, coupled with innovative low threshold models of care, as well as social-structural interventions addressing the underlying factors continuing to fuel the HCV epidemic, will be critical to maximizing the individual and population benefits of HCV treatment and move us closer to the 90-90-90 HCV target proposed by the WHO in September 2015 [10].

## References

[1] Shafran S. D. (2015). Hepatitis C and the sex trade. *Canadian Journal of Gastroenterology & Hepatology*.

[2] Socías M. E., Shannon K., Montaner J. S. (2015). Gaps in the hepatitis C continuum of care among sex workers in
Vancouver, British Columbia: implications for voluntary hepatitis C virus
testing, treatment and care. *Canadian Journal of Gastroenterology and Hepatology*.

[3] Feldman J. G., Minkoff H., Landesman S., Dehovitz J. (2000). Heterosexual transmission of hepatitis C, hepatitis B, and HIV-1
in a sample of inner-city women. *Sexually Transmitted Diseases*.

[4] Shannon K., Strathdee S. A., Goldenberg S. M. (2015). Global epidemiology of HIV among female sex workers: influence of
structural determinants. *The Lancet*.

[5] Shah H. A., Heathcote J., Feld J. J. (2013). A Canadian screening program for hepatitis C: is now the
time?. *Canadian Medical Association Journal*.

[6] Uto H., Mawatari S., Kumagai K., Ido A., Tsubouchi H. (2012). Clinical features of hepatitis C virus carriers with persistently
normal alanine aminotransferase levels. *Hepatitis Monthly*.

[7] Deering K. N., Montaner J. S., Chettiar J. (2015). Successes and gaps in uptake of regular, voluntary HIV testing
for hidden street- and off-street sex workers in Vancouver,
Canada. *AIDS Care*.

[8] Marmot M., Allen J. J. (2014). Social determinants of health equity. *American Journal of Public Health*.

[9] Grebely J., Robaeys G., Bruggmann P. (2015). Recommendations for the management of hepatitis C virus infection
among people who inject drugs. *The International Journal of Drug Policy*.

[10] WHO (2015). *Draft Global Health Sector Strategy on Viral Hepatitis,
2016–2020*.

